# Study protocol of “Our Choice”: a randomized controlled trial of the integration of safer conception counseling to transform HIV family planning services in Uganda

**DOI:** 10.1186/s13012-018-0793-y

**Published:** 2018-08-14

**Authors:** Kathy Goggin, Emily A. Hurley, Jolly Beyeza-Kashesya, Violet Gwokyalya, Sarah Finocchario-Kessler, Josephine Birungi, Deborah Mindry, Rhoda K. Wanyenze, Glenn J. Wagner

**Affiliations:** 1 Health Services and Outcomes Research, Children’s Mercy Hospitals and Clinics, Kansas City, MO USA; 2 Schools of Medicine and Pharmacy, University of Missouri–Kansas City, Kansas City, MO USA; 3 Department of Obstetrics and Gynaecology, Mulago Hospital, Kampala, Uganda; 4 Makerere University College of Health Sciences, Kampala, Uganda; 5 Department of Disease Control and Environmental Health, Makerere University School of Public Health, Kampala, Uganda; 6 Department of Family Medicine, University of Kansas Medical Center, Kansas City, KS USA; 7 The AIDS Support Organization, Kampala, Uganda; 8 Medical Research Council/Uganda Virus Research Institute, Entebbe, Uganda; 9 Los Angeles Center for Social Medicine and Humanities, University of California, Los Angeles, CA USA; 10 RAND Corporation, Santa Monica, CA USA

**Keywords:** HIV/AIDS, Safer conception methods, Safer conception counseling, Sub-Saharan Africa, Implementation science, Pregnancy, HIV prevention, Serodiscordant, Mother-to-child transmission, Sexual transmission

## Abstract

**Background:**

About 40% of HIV-positive women in sub-Saharan Africa become pregnant post-diagnosis. Despite about half of their pregnancies being planned, safer conception methods (SCM) are underutilized among serodiscordant couples, partially due to the fact that safer conception counseling (SCC) has not been integrated into routine HIV family planning (FP) services.

**Methods:**

*Our Choice* is a comprehensive FP intervention that promotes unbiased childbearing consultations to ensure clients receive SCC or contraception services to achieve their desired reproductive goals. The intervention is theoretically grounded and has demonstrated preliminarily feasibility and acceptance through pilot testing. This three-arm cluster randomized controlled trial compares two implementation strategies for integrating *Our Choice* into routine FP services vs. usual care. Six sites in Uganda will be randomized to receive either (1) *Our Choice* intervention with enhanced training and supervision provided by study staff (SCC1), (2) *Our Choice* intervention implemented by the Ministry of Health’s standard approach to disseminating new services (SCC2), or (3) existing FP services (usual care). *Our Choice* and usual care FP services will be implemented simultaneously over a 30-month period. Sixty clients in serodiscordant relationships who express childbearing desires will be enrolled by a study coordinator at each site (*n* = 360) and followed for 12 months or post-pregnancy (once, if applicable). Analysis will compare intervention arms (SCC1 and SCC2) to usual care and then to each other (SCC1 vs. SCC2) on the primary outcome of correct use of either SCM (if trying to conceive) or dual contraception (if pregnancy is not desired). Secondary outcomes (i.e., pregnancy, use of prevention of mother-to-child transmission services, condom use, and partner seroconversion) and cost-effectiveness will also be examined.

**Discussion:**

Findings will provide critical information about the success of implementation models of varying intensity for integrating SCC into FP, thereby informing policy and resource allocation within and beyond Uganda.

**Trial registration:**

NCT03167879 ClinicalTrials.gov, Registered 30 May, 2017.

## Background

In Uganda, approximately 40% of HIV-positive women become pregnant post-HIV diagnosis [1, 2]. Despite the fact that approximately half of their pregnancies are planned [1], people living with HIV (PLHIV) in sub-Saharan Africa rarely receive counseling on established, effective methods for making conception safer. Existing family planning (FP) services focus almost exclusively on preventing unwanted pregnancies and mother-to-child transmission. For the 14–73% of PLHIV who desire children [1, 3–6], many of whom are in serodiscordant relationships, the absence of safer conception counseling (SCC) in FP represents a missed opportunity to limit risks of horizontal and vertical transmission associated with childbearing. Further, the fact that half of pregnancies among HIV-positive women are unplanned reveals a high unmet need for effective contraception services. Comprehensive FP services can be most effectively positioned to reduce risk of HIV transmission when they help PLHIV and their partners make informed childbearing decisions and use established, effective methods for either safely conceiving and delivering a child or preventing unwanted pregnancies. In Uganda, where up to 50% of PLHIV in relationships have an uninfected partner [7, 8], these services are critically needed.

Comprehensive FP services depend on open discussion between healthcare providers and clients regarding childbearing desires. However, research in Uganda and elsewhere in sub-Saharan Africa suggests that 60–80% of PLHIV with fertility intentions do not discuss childbearing desires with providers [9–12]. Although slowly changing, providers have historically discouraged or even condemned childbearing among PLHIV, citing risks such as seroconversion and infant mortality [9–12]. Clients are often reticent to discuss childbearing due to perceived provider stigma or internalized stigma [9, 10, 13, 14]. Even among providers who hold supportive attitudes toward PLHIV with childbearing desires, research suggests that most do not feel confident enough in SCC to be able to provide appropriate guidance [12, 15–17]. This gulf in communication represents a significant obstacle to providing clients with services consistent with their pregnancy decisions.

The safety and efficacy of safer conception methods (SCM) is well established in the literature, with original efficacy research reporting no horizontal transmissions [18]. When practiced in conjunction with consistent adherence to antiretroviral therapy (ART), timed unprotected intercourse (TUI) greatly reduces risk of horizontal transmission by limiting unprotected intercourse to a few select days when the woman is most fertile [18, 19]. In cases where a woman is positive and her male partner is negative, the couple can achieve pregnancy through manual self-insemination (MSI) without any risk of viral transmission [19]. National guidelines supporting SCM have been published in North American and European countries [20–22] as well as in South Africa [23]. Internationally, the right to conceive among PLHIV is recognized by the World Health Organization and guidelines for providing access to SCM through SCC are in development [24].

Despite established efficacy, the implementation of SCM services in sub-Saharan Africa has lagged, meaning few serodiscordant couples who wish to conceive are practicing these methods [25–27]. While SCM cost little to nothing, our 24-month observational cohort study in Uganda revealed that only 15% of couples wishing to conceive reported using TUI and only 1% reported MSI at baseline [28]. At 24 months, self-reported rates of TUI increased to 35% with 1 and 9% reporting use of MSI and pre-exposure prophylaxis (PrEP), respectively [27]. Recent studies have shown that couples are receptive to using SCM and desire access to SCC, but education and outreach efforts are needed to increase knowledge of methods and address fears related to both risks of childbearing and use of “unnatural” conception methods [11, 29, 30].

Integrating SCC to FP may also help close existing gaps in contraceptive services and prevention of mother-to-child transmission (PMTCT). Nearly half of pregnancies among PLHIV in Uganda are unplanned, as only 15% of PLHIV use modern contraceptives other than condoms and only 11% use dual methods (condoms plus a second contraceptive) [1]. Further, one in five new HIV cases in Uganda result from vertical transmission [31], as 28% of HIV-infected pregnant women do not receive PMTCT [32]. Of those who do receive PMTCT, 63% do not fully adhere to the full continuum of care [33–36]. Periodic nonjudgmental, autonomy-respecting discussions about childbearing would enable clients to make more informed decisions about their reproductive health and providers to match services to client needs. For example, after couples considered the risks and benefits of childbearing through our pilot SCC intervention, about half opted not to pursue childbearing, opening the door for providers to guide them in choosing and utilizing effective contraception [37]. For couples that do pursue and succeed in pregnancy, SCC can provide early PMTCT counseling and linkage to services.

On the basis of promoting both human rights and public health, the World Health Organization and other international entities have made recent appeals to provide PLHIV with access to SCC [24]. While support for SCC is growing, evidenced-based implementation guidance is lacking. We recently conducted a pilot study of a comprehensive FP intervention consisting of patient-centered childbearing consultations to ensure that clients receive quality services consistent with their desired reproductive goals [37]. Findings demonstrated that such programs are acceptable to providers and clients and feasible to integrate into existing HIV services [37]. Nevertheless, rigorous implementation research is needed to guide large-scale efforts to integrate SCC and comprehensive family planning.

This protocol paper describes a parallel, cluster randomized controlled trial comparing two different implementation models for integrating SCC into the existing standard of care FP services in HIV clinics in Uganda. Specifically, six sites will be randomized to implement: (1) a comprehensive FP program that incorporates a structured, multi-component SCC intervention with training and supervision provided by study team members (SCC1) versus (2) the same FP/SCC program with trainings and supervision provided by Ugandan Ministry of Health (MoH) staff consistent with the routine roll out of new programs (SCC2) versus (3) existing FP services (usual care). The primary outcome will be use of either SCM (for those who continue to try to conceive after consultation) or dual contraception (for those who chose to prevent or delay pregnancy after consultation). Impact on secondary outcomes of pregnancy, PMTCT use, condom use, and partner seroconversion will also be examined. Potential moderators and mediators of any intervention effects on SCM and contraception use will be explored. The cost-effectiveness of the new comprehensive FP intervention (SCC1 and SCC2) will be compared to usual care. The overarching goal of this study is to inform MoH policy and resource allocation.

## Methods

### Setting and participants

The study will be conducted at six HIV clinics operated by The AIDS Support Organization (TASO). TASO is the oldest and one of the largest indigenous non-governmental organizations in Uganda providing comprehensive HIV prevention, care, and support services. Each site resides within a hospital campus, provides HIV care to 6000–8000 index clients (65–75% female, 75–80% aged 15–49, ~ 70–80% on ART), and has a staff of 15–20 medical providers (including 3–4 FP nurses), 12–15 counselors, and 6–10 expert clients. TASO provides family planning, contraception, and PMTCT services, but has yet to integrate safer conception services.

Each study site will implement their assigned FP program (SCC1, SCC2, or usual care) clinic-wide for all clients. To evaluate the impact of the services, we will enroll 60 clients at each site (120 per arm for total of 360 clients) who meet the following eligibility criteria: (1) in a serodiscordant relationship (partner’s HIV-negative status confirmed by rapid HIV test prior to enrollment), (2) of reproductive age (men age 15–60; women age 15–45), (3) considering childbearing with their spouse/partner (determined via triage screening item), (4) not currently pregnant (determined by a pregnancy test for female partner prior to enrollment), and (5) reports having disclosed HIV status to partner. Recruitment will be stratified by sex to ensure a 50/50 gender balance, which will better enable us to examine intervention effects on partner seroconversion and to gain the perspective of both male and female clients regarding fertility. To reach the target sample size over the 18 months of the study, we plan to enroll 3–4 clients per month at each site. In order to improve our ability to retain participants in the study, we will collect multiple contact phone numbers, location of home, name and contact numbers for family members and/or friends who would be able to contact the client if they move or are away, and name of a TASO expert client or other community health care worker that they know and trust. We have successfully used these strategies in other studies where we have seen low levels of attrition.

### Randomization

A blind drawing witnessed by the leadership of each clinic site will be used to randomly assign sites to one of the two implementation models for integrating SCC into FP services (SCC1 or SCC2) or usual care (existing FP services).

### *Our Choice* SCC intervention

**Fig. 1:**
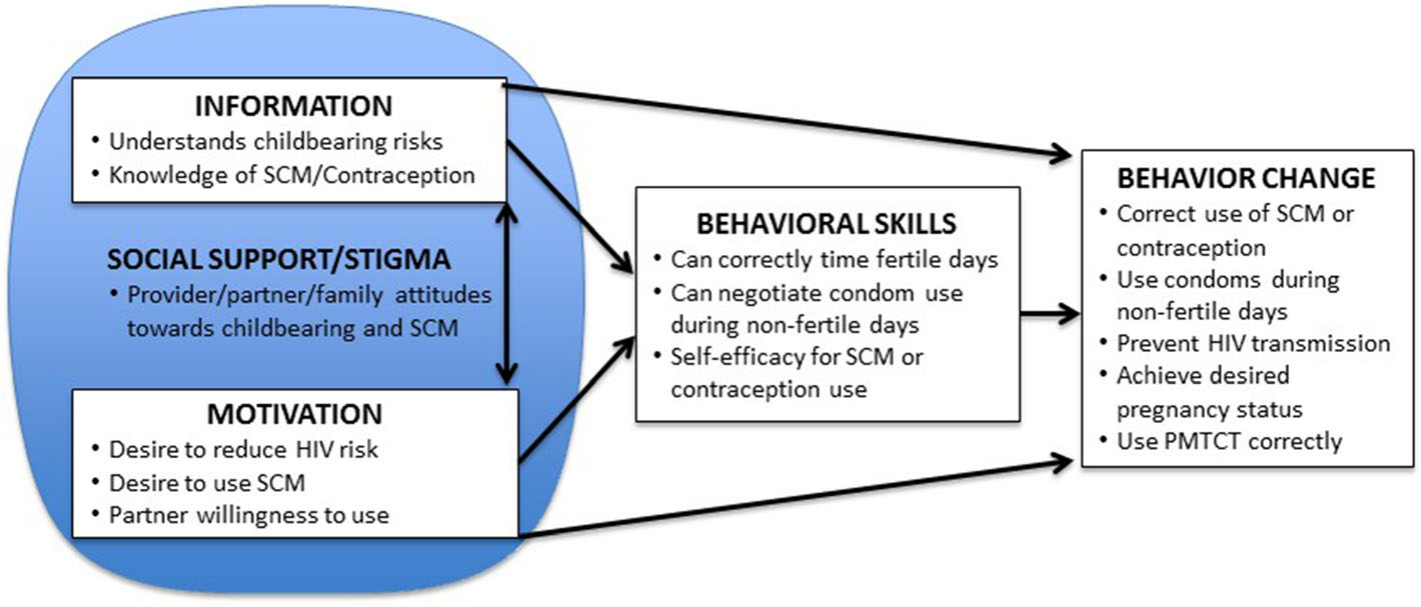
“Our Choice” intervention targets for each construct of the ecological information, motivation, and behavioral skills (eIMB) model

Informed by our earlier research [11, 15, 17, 27, 35, 38] and guided by an ecological adaptation of the information, motivation, and behavioral skills (eIMB) model of behavior change [39], we developed a multi-component, structured intervention that identifies and engages HIV clients and their partners with fertility desires in SCC. The goal of the *Our Choice* intervention is for providers to facilitate an informed decision process regarding childbearing that supports each couple’s decision with training on the use of contraception or SCM (and PMTCT once pregnant) in accordance with their decision. Figure 1 illustrates the client and partner intervention targets for each eIMB construct. The intervention consists of three major components: (1) client and community outreach, (2) routine screening of childbearing desires at triage, and (3) provision of structured, manualized SCC sessions.

**Fig. 2:**
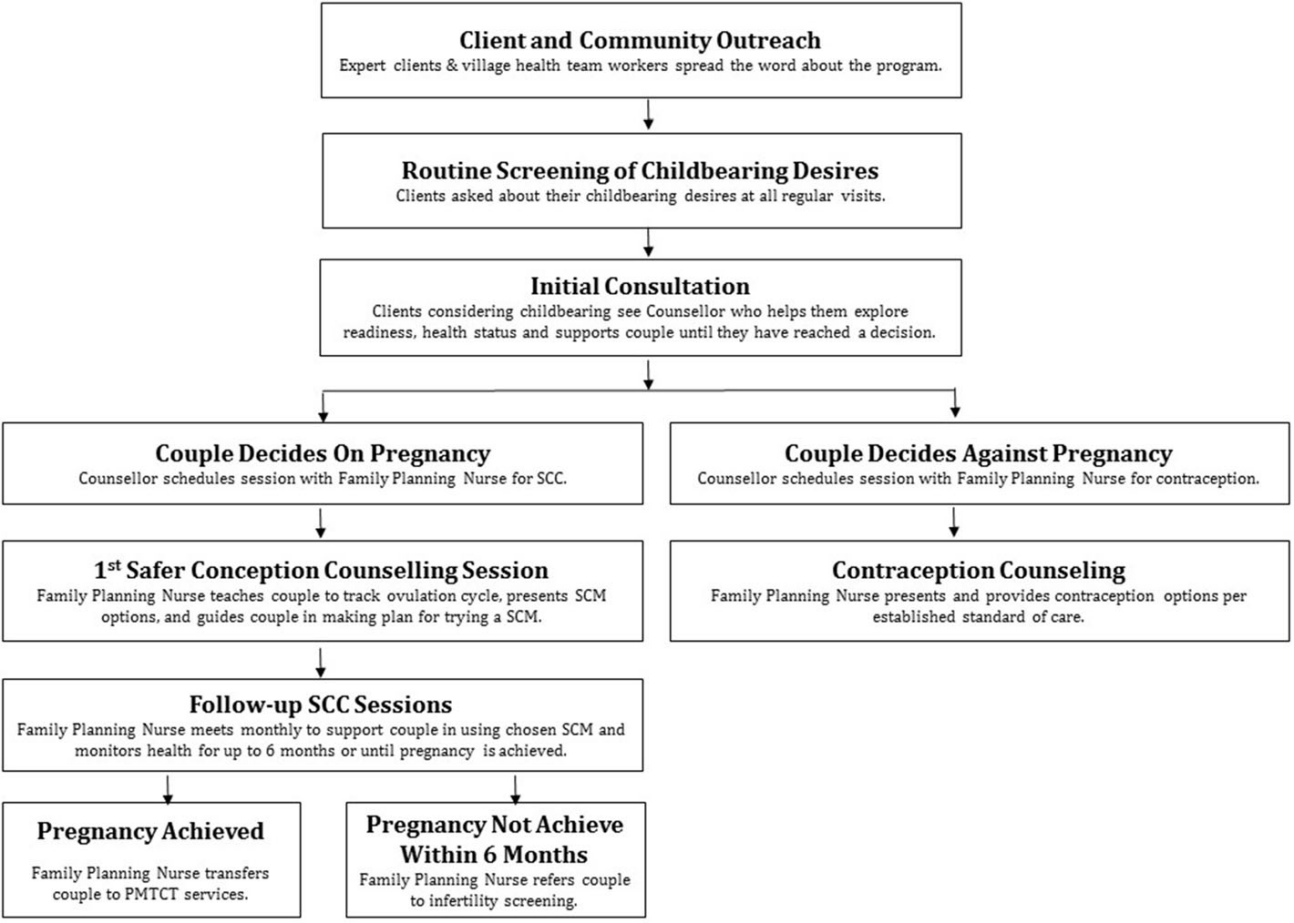
“Our Choice” safer conception and contraception counseling intervention flow diagram

*Client and community outreach:* Expert clients and village health team workers (VHTs) will engage PLHIV in their communities with the goal of promoting knowledge about SCM and contraceptive services at TASO, reducing childbearing stigma and identifying interested PLHIV and their partners who they will direct to services. *Routine screening of childbearing desires***:** Clients checking into their regular visits will be asked a single screening question, “Are you thinking about trying to conceive a child within the next 12 months?” by the expert clients at triage. Clients who report childbearing desires will be seen by a trained counselor for an initial SCC consult during the same clinic visit or scheduled for a future visit if the client is willing and able to bring their partner in. Counselors will work with clients until they make an informed decision about whether they want to try to conceive or not. Couples who decide against trying to conceive at this point, or at any time in the counseling, will be referred to trained FP nurses for contraception. Couples who decide to try to conceive will be referred to trained FP nurses for further SCC (Fig. 2). *Provision of structured*, *manualized SCC sessions*: Using a structured protocol and comprehensive manual, trained FP nurses will provide up to 6 monthly SCC sessions to promote informed childbearing decisions and use of SCM, PMTCT, and contraception.

**Fig. 3:**
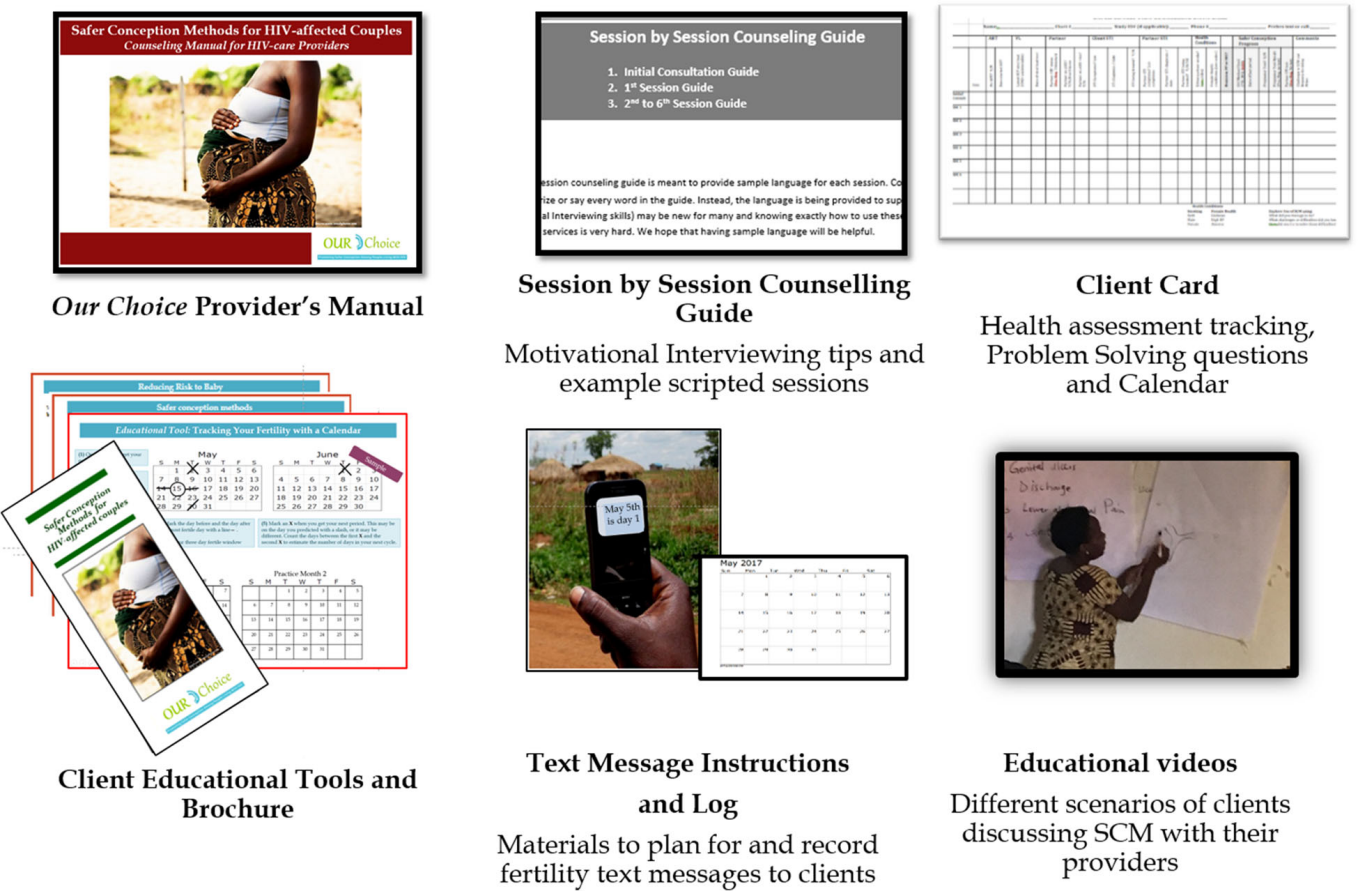
“Our Choice” intervention materials

The sessions and manual were developed and improved through serial pilot testing and include guidance on autonomy supportive decision-making, motivational interviewing (MI) counseling skills, engaging partners, fertility tracking and SCM, health considerations and risk reduction, and linkages to contraceptive or PMTCT services. Topics addressed in each session are summarized in Table 1, and intervention materials are pictured in Fig. 3. In addition to the manual, providers will be given tools to facilitate SCC including session checklists, pregnancy planning calendars, client educational materials, client text message log, and client SCM kits (including needleless syringe, plastic cup, non-spermicidal condoms, and lubricants). The intervention will be disseminated to the active arms (SCC1 and SCC2) using different implementation models described below. No intervention materials or staff trainings will be offered at the usual care sites.

**Table 1:** Safer Conception Counseling Topics by Session

Initial Consultation (45–60 min)	* Build rapport, explain services, communicate non-judgmental support for couple’s decisions
	* Explore contextual issues (i.e., client’s childbearing interest, partner’s childbearing interest, partner’s HIV status, family support, disclosure, existing children, health of relationship, available resources, planned separations due to work).
	* Review HIV and health risks of childbearing for mother/infant/partner and factors impacting risk (i.e., health, SCM, ART, CD4 cell count, PMTCT, STIs, alcohol use, nutrition). Encourage delaying pregnancy if medical condition not optimal (e.g., not on ART > 6 months, CD4 < 200, active STI) and provide treatment (for STIs or ART) as needed.
	* Introduce safer conception methods.
	* Encourage couple to take time to decide and return for SCC or contraception.
SCC Session 1 (20–30 min)	* Review couple’s fertility decision. Provide contraception if no longer desire a child.
	* Teach couple to track woman’s ovulation cycle using educational tools.
	* Present SCM using educational tools and assist couple to select their best method. Share videos, offer tools, MSI kit, offer text messages to remind client of start of fertile period.
	* Discuss other risk reduction options (i.e., circumcision, sperm washing, and PrEP).
	* Develop action plan with couple.
Follow-up Sessions (20 min)	* Review couple’s successes and challenges with action plan using Problem Solving worksheet.
	* Assess usefulness of tools, text messages and identify strategies to overcome barriers.
	* Assess HIV-positive’s partner’s ART adherence and refer for adherence counseling if needed.
	* Adjust action plan as needed; assess for STIs and treat as needed.
	* If partner is not attending sessions or hindering use of SCM, discuss strategies for addressing.
	* If woman’s period is late, conduct pregnancy test. If pregnant, conduct HIV testing with partner and start HIV-positive mothers on PMTCT.
	* After 6 months of correct SCM use, if pregnancy has not been achieved, discontinued SCC and refer couple to infertility clinic.

### Implementation models

Consistent with the goal of the study to compare different implementation models, SCC1 and SCC2 will use different approaches to implementing the SCC intervention. The major differences between the two implementation models will be the time and method of training and supervision of providers. Informed by our prior research [15, 37, 40], SCC1’s implementation model was designed to provide intensive yet scalable training and supervision that increases providers’ skills and self-efficacy to provide SCC while addressing their own beliefs and concerns about promoting fertility decision-making among PLHIV. The SCC2 implementation model was designed to reflect the normal MoH process for integrating new treatment advances into routine care and represents a highly viable, less intensive alternative to the training and supervision provided in SCC1. Consistent with the normal practice of rolling out new services, the study team will provide a full day “training of trainers” for MoH trainers/supervisors on the *Our Choice* program and provide copies of all manuals, client educational tools, educational videos, and SCM tool kits. Per usual practice, the MoH training team will then create their own clinical training program, continuing education, and supervision schedule and will independently organize, conduct, and oversee all aspects of services provided in the SCC2 arm. The usual care sites will continue to provide FP services as is, with no use of routine screening of childbearing desires or SCC. A one hour overview of the study will be conducted, but no training or supervision will be provided at these sites. Routine HIV FP care in Uganda is unlikely to incorporate SCC during the course of this study, as SCC guidelines have been available for years, yet SCC has remained scarce. Also, TASO has indicated it has no plans to integrate SCC into usual care until the study ends and efficacy and cost-effectiveness data can be reviewed.

### Provider training and supervision

#### Expert client training and supervision

In both SCC1 and SCC2 arms, expert clients will receive a full day training on client and community outreach, childbearing needs of PLHIV, and availability of comprehensive FP services including SCC and contraception. Topics covered will include PLHIV fertility rights, risks of pregnancy, issues couples should consider before attempting to get pregnant, methods to reduce risk and improve mother/child health, aspects of a home visit, and mobilizing clients. An accompanying manual addressing all of these topics will be provided. Training will stress that detailed information about SCM should be given by TASO counselors and FP nurses, not expert clients. In SCC1 sites, training will include an additional focus on expert clients’ own beliefs and concerns about PLHIV having children, their role in promoting use of SCM and an introduction to the autonomy-supportive counseling skills that their fellow TASO providers will be using during SCC. No training will be provided in control sites. All SCC1 and SCC2 expert clients will be provided with 10,000 Shillings (~$2.80) for every serodiscordant couple that they bring to the study.

#### Counselor and FP nurse training and supervision

Counselors and FP nurses will be guided to develop the core skills detailed in Table 2 by employing the training, supervision, and fidelity strategies listed in columns three and five.

**Table 2:** Target skills, training, supervision and fidelity checks for counselors and family planning nurses, by intervention arm

	Skills	Training Strategy	Hours	Supervision & Fidelity
SCC1: Study team-led implementation model	− Building rapport	− Exploration of counselors’ beliefs and attitudes	16	− Supervision ▪ Semimonthly individual and group; ▪ Role-plays
	− Exploring readiness ▪ Using open question ▪ Using reflective listening ▪ Offering information			
		− Didactic review of manual		
		− Videos / live demos		− Sessions recorded for coding & tailored feedback
		− Role plays ▪ Communication skills ▪ Intervention sessions ▪ Teaching use of tools		
				− Session checklists ▪ Counselor completes ▪ Supervisor provides tailored feedback on coverage of content and MI skills
	− Assessing health factors			
	− Assisting w/ ▪ disclosure ▪ family planning decision			
		− Personalized feedback: MI skills & content mastery		
		− Identification of implementation barriers and solutions		
	− Confidence providing SCC			
				− 95% of text messages sent on time
	− Teaching SCM and use of tools	− Hands-on text training		
	− Problem solving strategies			
	− Using text message system			
SCC2: MoH-led implementation model	− Knowledge of SCM	− Lecture on benefit of SCC and history of stigma	8	− Supervision on request
	− Confidence providing SCC			
		− Read manual sections Answer provider questions		− Quarterly MoH support visits
	− Familiar w/ manual and tools			
				− Yearly training update
		− Videos / live demos		
		− Practice tools		

The major differences between the SCC1 training and supervision model from that which will be offered in SCC2 will be additional time and attention to reduce SCC stigma among providers and develop their ability to use autonomy-supportive counseling skills. Guided by our previous research [15, 40], we will use active learning approaches in SCC1 training to explore the source of provider stigma, as well as providers’ own beliefs and concerns about providing SCC. Through high quality training and ongoing supervision support, we will also develop and sustain providers’ skills and self-efficacy for providing SCC. In addition, we will enhance providers’ autonomy-supportive counseling skills by training them in three essential motivational interviewing skills (i.e., open questions, reflective listening, and offering advice). SCC1 providers will receive two days of training and ongoing supervision from trained study staff. SCC1 supervisors will visit sites twice monthly for the first six months and then once monthly throughout the study. SCC1 providers will be encouraged to share tapes of their SCC sessions, so supervisors can provide tailored feedback and support continued skill development. In contrast, provider training in SCC2 sites will occur over one day, and MoH supervisors will conduct supervision according to their schedule, which has typically been quarterly when other new services have been integrated into usual care.

### Measures

#### Assessment procedures

The baseline assessment will be done by the site coordinator once eligibility is confirmed (i.e., partner tests HIV-negative; female partner has a negative pregnancy test), followed by assessments at months 6, 12, and post-pregnancy (if pregnancy occurs prior to month 12). Assessments will include measures of the primary and secondary outcomes and variables that map to our eIMB conceptual framework for understanding use of SCM and contraception, as listed below. Survey measures have under gone translation, back-translation, and review process in Luganda and Runyakitara. The survey will be interviewer-administered using computer-assisted software, and administration time is estimated at 45–60 min.

#### Primary outcome

The primary outcome is correct use of contraception or SCM over the past 6 months, depending on pregnancy intentions, as a single binary indicator. Pregnancy intentions can change, so the primary outcome will be defined as concordance between correct use of contraception or SCM consistent with couples’ intentions during the assessment period. Continuous and categorical measures of frequency and duration of contraception/SCM use will be used in secondary analyses. Consistent with FP policy and practice in Uganda, correct use of contraception is defined as always using dual contraception, or both condoms and another modern contraceptive (e.g., oral pills, DEPO injection, IUD, implant) over the past 6 months (since last survey). Contraception use will be ascertained via self-report and chart review (provision of non-condom contraceptive). Male participants will be requested (not required) to consent to the coordinator calling their partner to assess use of non-condom contraceptives. Data will not be shared with the male partner. If a participant becomes single during the course of the study, we will conduct a final assessment and refer to a FP nurse for contraception counseling. To assess correct SCM use, respondents will be asked if they used TUI, MSI, or sperm washing in the past 6 months and how often. Given the cost of sperm washing, TUI and MSI are the two feasible methods for most clients. Clients will be asked in an open-ended format to describe exactly how they implemented the method. For each method, the interviewer will be looking for 3–5 pre-established pieces of information from the participant’s response (e.g., identifying the timing of woman’s fertile period; identifying number of days in the fertile period; having unprotected sex during the fertile period; using condoms outside of the fertile period; collecting and inserting semen using a syringe). Based on the information offered in the participant’s response, the interviewer will rate the use of the method as “fully accurate”, “inaccurate” or “no use”.

#### Secondary outcomes

Secondary outcomes include (1) achievement of desired pregnancy status and pregnancy outcome (live birth or miscarriage assessed via self-report and chart abstraction); (2) partner seroconversion (assessed by HIV testing at month 12); (3) PMTCT adherence (enrollment by 14 weeks gestation, ART use during pregnancy and post-delivery, infant ART prophylaxis, and early infant diagnosis of HIV) for those who become pregnant (assessed via chart abstraction); (4) self-reported consistent condom use in past 6 months (generally for those not seeking childbearing, and all times outside of the 3-day fertile period of each month for those seeking childbearing and using TUI/MSI).

#### Mediators/moderators of primary and secondary outcomes

Potential mediators (e.g., information, motivation, behavioral skills, socio-cultural factors) and moderators (e.g., demographics, reproductive history, HIV/medical characteristics, partner/relationship characteristics) of outcomes will be included in the assessments. All variables are drawn from our eIMB framework, and all are adapted versions of established measures or items/scales that were developed and validated by our team [41].

#### Process evaluation and fidelity monitoring

To assess clinic-level FP capacity, fidelity to FP guidelines and the *Our Choice* intervention, and to inform data interpretation and sustainability, we will (1) interview clinic administrators, counselors/FP nurses, and expert clients at all sites and clients at SCC1 and SCC2 sites at baseline, 12 and 24 months; (2) analyze routinely collected clinic FP data; and (3) use FP services data from participant surveys to triangulate with the nurse/administrator interview data.

##### Interviews

Clinic administrator interviews will focus on successes and challenges in providing FP/SCC services and the influence of structural barriers. Counselor/FP nurse interviews will focus on successes and challenges in engaging clients and their partners in FP/SCC, making fertility decisions, and understanding and using SCM; client follow-up; partner engagement; and the influence of cultural and structural barriers. We will also explore what aspects of the intervention are working better than others and conduct role plays at study end to assess counselor/FP nurses’ content knowledge and motivational interviewing communication skills. Expert client interviews will focus on the content of their messaging regarding availability of SCM and SCC, potential for reducing risk, and the importance of communicating with providers and partners. In SCC1 sites, we will compare interview responses over time to assess for change in quality with ongoing training and supervision and use of motivational interviewing communication skills. We will also ask about client and community responses to their outreach, observed resistance, and how they responded to resistance. Client interviews (representing clients seeking and not seeking children) will explore experiences with FP/SCC services, partner engagement in SCC, actual use of SCM/contraception, and suggestions for how to improve services. Expert clients will be asked to keep a log of their outreach activities, including group talks in the clinic waiting room or community and one-on-one discussions, both in terms of when an activity occurred and the number of people present. These data will be complemented by session checklists completed by FP nurses.

##### Routinely collected clinic FP data

This data will be used to analyze FP services in the overall clinic population to examine clinic capacity and fidelity to the intervention. The triage book and childbearing screening item (in SCC1/SCC2 sites) will be used to determine the following: (a) number of participant who do not want children and percentage of those who receive contraception services, and (b) number of participants who report childbearing desires. FP registries will indicate the number of new clients receiving FP services (not seen by FP in past year) and the percentage of FP recipients using dual contraception. These will be tracked longitudinally to assess intervention effects on clinic-level FP services. The percentage of clients receiving FP referrals after triage will be compared across all three arms using research cohort data since childbearing screening is not used at the usual care site.

##### Triangulate participant survey data with the expert client/counselor/nurse/administrator interview data

This will be accomplished by comparing emergent themes found in the qualitative analysis of the interview data with participants’ survey responses.

### Data analysis

#### Power and sample size

In Uganda, 11% of PLHIV in care who want to prevent pregnancy use dual contraception [1] and our research shows 15% of PLHIV trying to conceive use TUI or MSI [28]. Therefore, we expect the usual care arm will have a 15% rate of correct use of contraception/SCM (depending on pregnancy intentions), which is the primary outcome. Using an intra-class correlation (ICC) of 0.01 to control for clustering, based on other studies of reproductive health outcomes [42], and 10% attrition at month 12 (given the 6% in our pilot study), we will have > 80% power (two-tailed test) to detect a 6% point difference (small effect size; *d* = 0.15) for our comparison of the usual care arm to the combined SCC1 and SCC2 arms on the use of contraception or SCM at month 12, and a 9% point difference between the SCC1 and SCC2 intervention arms. In analysis of effects on contraception and SCM use separately, and with half the sample expected to decide to pursue pregnancy and half contraception based on our pilot data, our sample size (*n* = 360) will provide > 80% power to detect a 6.5% point difference with regard to each of the outcomes when comparing usual care to SCC1 and SCC2 combined, and 8–9% point difference between the SCC1 and SCC2 interventions.

#### Analysis of primary and secondary outcomes

Our primary analysis will be to compare the combined intervention arms (SCC1 and SCC2) against the usual care control to assess the effects of integrating SCC into FP services and then compare outcomes between the two intervention arms to determine whether the more intensive SCC1 shows a larger effect than the less intensive SCC2. We will use an intent-to-treat approach in the primary analyses; secondary analyses will use only study completers. Attrition weights will be used to account for dropouts, and analyses will incorporate design effects from this weighting in the calculation of standard errors and tests of significance. We will account for clustering, and with only six clusters/clinics, we will conduct individual-level analysis. Regression methods can directly model correlations among units in a cluster (random effects or multilevel models) or more simply adjust standard errors for clustering (GEE, robust standard errors), but these approaches are not reliable with so few clusters [43]. We will use standard regression methods, but rather than attempt to estimate ICC directly, we will explore the sensitivity of significance levels using a range of plausible ICCs values for the outcomes.

The statistical analyses will aim to:

1. Assess intervention effect on contraception/SCM use: We will test the hypothesis that both SCC1 and SCC2 will be superior to usual care, and SCC1 will be superior to SCC2 on the primary outcome. We will perform logistic regression to compare rates of contraception or SCM use (depending on pregnancy intention) in the SCC1 and SCC2 interventions compared to the usual care condition and investigate differences between SCC1 and SCC2 in a separate regression. The models will include covariates to adjust for baseline variables (e.g., childbearing stigma, age, sex) that may differ at baseline and to increase precision of the estimates.
2. Assess intervention effect on secondary outcomes: We will use the same analytic strategy as described in the previous aim to compare the three arms on the secondary outcomes of pregnancy status, PMTCT use, consistent condom use, and partner seroconversion.
3. Examine eIMB moderators and mediators of intervention effects on SCM and contraception use: We will identify bivariate correlates of the primary outcome from among baseline variables using correlation coefficients, *t*-tests, and chi-square tests, followed by a stepwise regression approach to identify a parsimonious list of predictors. We will then use longitudinal analyses to investigate changes in the primary outcome relative to changes in dependent variables such as SCM knowledge, motivation, and self-efficacy. Like the baseline cross-sectional analysis described above, we will start with bivariate analyses that will inform a subsequent stepwise regression approach. We will use clinic-fixed effects because with only six clinics, clustering standard errors to correct for correlation between patients in the same clinic would significantly reduce power. Robust estimation techniques can adjust for correlation in multiple observations of a given patient over time. We will use a similar analytic approach to examine determinants of the secondary outcomes. Lastly, we will investigate the paths through which the intervention works and subgroups that benefit more from the intervention by adding selected interaction terms. Using methods described by MacKinnon [44] and others [45, 46], we will assess whether the intervention has a direct impact on contraception/SCM use or mostly though a mediating variable such as communication with provider about childbearing needs (which may result in clients being more apt to receive the information and encouragement needed to use SCM/contraception) [47, 48].

#### Qualitative data analysis

Qualitative interviews will be audio-recorded, transcribed, and translated into English. Data will be entered in Atlas-ti [49], organized thematically with a codebook, and coded by two team members using a grounded theory approach. Inter-coder reliability will be assessed and consensus reached where there is disagreement [50]. Topical codes will be used to index interviews. Results will be aggregated to identify common themes and patterns across participants [51, 52].

#### Cost-effectiveness analysis

We will track all clinic costs associated with implementing SCC1 and SCC2, including costs of existing FP services, and additional expenses such as labor costs associated with SCC sessions and consults with FP nurses, supervision of the FP nurses (in SCC1), contraceptives and SCM client kits, and intervention materials. Labor costs are based on clinic-specific data, external sources, and documentation of SCC sessions, FP consults, and supervision meetings. Fixed costs will be allocated to the intervention as the fraction of time the premises are used for the intervention (e.g., SCC sessions). Training materials and supplies will be costed at purchase prices. Development costs such as personnel costs for the SCC training workshop will be differentiated from ongoing operational costs, but will exclude research costs (e.g., surveys). We will also evaluate potential efficiencies in operational costs (SCC sessions and ongoing supervision) over time and differentiate between the fixed costs of the intervention and the marginal cost of adding an additional client to inform generalizability to other settings. We will assess whether the intervention is cost-effective by assessing the cost-effectiveness ratio, defined by the difference in per-capita cost of the two intervention models relative to the control. We will assess average overall and marginal cost per achieved pregnancy (without partner seroconversion) or prevented pregnancy (depending on the client’s desired pregnancy status) and compare those to published values such as Shade et al. [53]. We will estimate confidence intervals for our cost-effectiveness ratios using bootstrap methods [54].

### Monitoring

An independent monitor with appropriate scientific and local Ugandan expertise will meet regularly (twice per year) with study team. The independent monitor will have no relationship to the sponsor, have no competing interests, and develop the charter (available upon request from the study team). During regularly scheduled meetings with the study team, the independent monitor will review the study protocol, materials, planned analyses, and procedures for protecting participants’ confidentiality and reducing risk, adverse events reporting protocol, and summary data of recruitment, retention, outcomes, and adverse events by arm. The independent monitor will issue a written report after meetings detailing any necessary protocol changes and/or closing of the study as outlined in the charter. No interim analyses are planned. The adverse event protocol calls for regular reporting of adverse and unexpected events experienced by participants to study coordinators during bi-monthly data collection. Likelihood of adverse events being related to study participation will be determined by the TASO medical staff in consultation with the study team. All adverse events are reported to the Institutional Review Boards (IRBs) in accordance with their required timelines and detailed in the adverse event protocol. Beyond regular reviews conducted by the independent monitor, the Makerere School of Public Health Research and Ethics Committee conducts regular audits of approved human subject research studies to ensure compliance with the study protocol and IRB requirements.

### Ethics and dissemination

Our protocol has been approved by the IRBs at Makerere School of Public Health and the RAND Corporation. Any modifications to this protocol will be submitted for IRB approval and communicated to all relevant parties prior to implementation. Informed consent will be administered by trained study staff and will be required prior to participation. All research data will be kept in locked file cabinets or encrypted password-protected electronic files and will be available only to research staff directly involved in this project and regulatory agency personnel. Any audio-recordings will be stored on encrypted computer files and securely deleted once reviewed by supervisors (within two weeks of recording). Data will be identifiable only by study numbers and patient initials. HIV status will not appear along with any personal identifying information. Personal information including subjects’ name, address, and phone number will be stored separately from all research data. Participants will be eligible for care from TASO and government provided health services during and after the study.

Results of this study will be presented at local and international conferences and submitted for publication in peer-reviewed journals. We will adhere to authorship criteria outlined by each journal to determine author eligibility. Only members of the study team will access to the final trial dataset which will be locked and access controlled by the data analysis study team members. A full data package will be maintained by the study investigators for at least seven years after data collection is complete. Third-party access to the full data package will be addressed by the study team on a case-by-case basis.

## Discussion

Comprehensive family planning services have the potential to optimize prevention of horizontal and vertical transmission of HIV by helping PLHIV prevent unwanted pregnancies, use SCM when they desire pregnancy, and engage in PMTCT when they become pregnant. This study seeks to evaluate and establish an approach for comprehensive family planning services for PLHIV that integrates routine childbearing discussions and safer conception counseling with existing contraception services (Fig. 2). Recognizing the need for a model that is efficacious as well as scalable and sustainable, the proposed study will not just examine one intervention, but two models of SCC integration that differ in their intensity level and perform a cost-effectiveness analysis. Results will inform recommendations for policy and practice across the range of resource levels present in Uganda and sub-Saharan Africa.
